# A systematic review and meta-analysis of preclinical trials testing anti-toxin therapies for *B*. *anthracis *infection: A need for more robust study designs and results

**DOI:** 10.1371/journal.pone.0182879

**Published:** 2017-08-10

**Authors:** Wanying Xu, Lernik Ohanjandian, Junfeng Sun, Xizhong Cui, Dante Suffredini, Yan Li, Judith Welsh, Peter Q. Eichacker

**Affiliations:** 1 Critical Care Medicine Department, Clinical Center, National Institutes of Health, Bethesda, Maryland, United States of America; 2 National Institutes of Health Library, National Institutes of Health, Bethesda, Maryland, United States of America; New York State Department of Health, UNITED STATES

## Abstract

**Background:**

*B*. *anthracis* anti-toxin agents are approved and included in the Strategic National Stockpile based primarily on animal infection trials. However, in the only anthrax outbreak an approved anti-toxin agent was administered in, survival did not differ comparing recipients and non-recipients, although recipients appeared sicker.

**Objective:**

Employ a systematic review and meta-analysis to investigate preclinical studies supporting anthrax anti-toxin agents.

**Data source:**

PubMed, EMBASE, and Scopus.

**Study eligibility:**

Compared survival with an anti-toxin agent versus control in *B*. *anthracis* challenged, antibiotic treated animals.

**Study methods:**

Examine model and study design and the effect of anti-toxin agents on relative risk of death(95%CI) (RR).

**Results:**

From 9 studies, 29 experiments were analyzed which included 4 species (748 animals) and 5 agents; LFI, AIG, AVP-21D9, Raxibacumab, and ETI-204. Only five experiments were blinded and no experiment included the cardiopulmonary support sick *B*. *anthracis* patients receive. Only one agent in a single un-blinded experiment reduced RR significantly [0.45(0.22,0.940]. However, in six studies testing an agent in more than one experiment in the same species, agents had consistent survival effects across experiments [I^2^ = 0, p≥0.55 in five and I^2^ = 42%, p = 0.16 in one]. Within each species, agents had effects on the side of benefit; in one study testing AVP-21D9 in mice [0.11(0.01,1.82)] or guinea pigs [0.70(0.48,1.03)]; across eight rabbit studies testing LFI, Raxibacumab, AIG or ETI-204 [0.62(0.45,0.87); I^2^ = 17.4%, p = 0.29]; and across three monkey studies testing Raxibacumab, AIG or ETI-204 [0.66(0.34,1.27); I^2^ = 25.3%, p = 0.26]. Across all agents and species, agents decreased RR [0.64(0.52,0.79); I^2^ = 5.3%, p = 0.39].

**Limitations:**

Incidence of selective reporting not identifiable.

**Conclusions:**

Although overall significant, individually anti-toxin agents had weak beneficial effects. Lack of study blinding and relevant clinical therapies further weakened studies. Although difficult, preclinical studies with more robust designs and results are warranted to justify the resources necessary to maintain anti-toxin agents in national stockpiles.

## Introduction

Due to the relative facility with which *B*, *anthracis* spores can be produced and widely delivered and the lethality of *B*. *anthracis* infection itself, this bacterium is considered a Biodefense Category A pathogen presenting a high risk to the US public.[1] This risk was highlighted by the outbreak of *B*. *anthracis* infection that occurred in the US in 2001 related to intentionally contaminated mail.[2,3] During that outbreak, 5 of 11 patients with inhalational disease died despite receiving aggressive therapy with antibiotics the bacteria was sensitive to, together with intensive supportive measures. Also, while the experience was small, mortality among those developing evidence of septic shock appeared much higher than with more commonly encountered bacteria. This outbreak along with other national and international events at the time, greatly accelerated research directed at developing adjunctive treatments that would add to or increase the efficacy of conventional ones for the treatment of *B*. *anthracis*. Because prior experience pointed to the production of lethal and edema toxins (LT and ET respectively) as central to the pathogenesis of *B*. *anthracis* infection, much of this research focused on developing agents to neutralize these toxins. Several potential therapies have resulted from this intensive research effort and the US Centers for Disease Control and Prevention (CDC) guidelines now recommend that patients with evidence of systemic and severe infection receive an anti-toxin agent combined with antibiotics and other supportive measures[4,5].

Three anti-toxin agents have now received US Food and Drug Administration (FDA) approval for use during an outbreak of anthrax infection including; anthrax immune globulin (AIG), a polyclonal antibody; Raxibacumab, a monoclonal antibody (mAb) and; and ETI-204, another monoclonal antibody.[6,7,8] Each of these agents is directed at inhibiting protective antigen (PA) mediated uptake of toxin by host cells. Because the naturally occurring invasive and highly lethal forms of *B*. *anthracis* infection are rare, these new agents have not been tested clinically. Instead, the FDA approved them based on the results of trials in animal models of *B*. *anthracis* infection under the animal rule together with safety studies in healthy humans. This experience has also provided the basis for including AIG and Raxibacumab in the Strategic National Stockpile (SNS) and for consideration of such inclusion for ETI-204. However, while these agents may be safe, whether they are efficacious in humans is unknown.

At this time there has only been one clinical experience with an approved anti-toxin agent during an outbreak of invasive *B*. *anthracis* infection. Anthrax immune globulin (AIG) was administered to 15 of 47 patients with *B*. *anthracis* soft-tissue infection during the 2009 to 2010 outbreak in Scotland related to the use of contaminated heroin.[9] Notably, review of 43 patients from that outbreak including all AIG recipients, showed that mortality in this uncontrolled experience did not differ comparing recipients and non-recipients. However, comparisons between these recipients and non-recipients are difficult to make. Related possibly to higher bacterial loads, more extensive tissue injury or underlying co-morbidities, patients receiving AIG had higher sequential organ failure scores and were sicker than non-recipients at presentation in this outbreak. Also, AIG was used off label for the soft tissue infections comprising this outbreak since all antitoxins licensed thus far, including AIG, have been developed for inhalational infection alone.

However, these findings from the UK outbreak combined with the substantial resources necessary to supply and maintain *B*. *anthracis* anti-toxin agents in the SNS, prompted us to try and better understand the strength of evidence derived from preclinical animal models of infection supporting such agents. To do this, we performed a systematic review and meta-analysis. We searched the literature looking specifically for preclinical trials that compared the effect of a *B*. *anthracis* anti-toxin agent to a control treatment in animal models employing live *B*. *anthracis* challenges. Because antibiotics are an effective therapy for *B*. *anthracis* infection if used early enough and are routinely administered to patients, we also required that the trials analyzed were conducted in antibiotic treated animal models. While a prior systematic review examined a similar question, the present investigation includes almost double the number of animals as this other study.[8] It also includes a meta-analysis of the retrieved studies, which the prior review did not.

## Materials and methods

### Literature search and study selection

PubMed, EMBASE, and Scopus databases were searched to retrieve relevant studies on the preclinical assessment of *B*. *anthracis* toxin associated inhibitors. The search was not limited by year or language and was conducted through July 31, 2016. Principle keywords included *B*. *anthracis*, *anthrax*, *antitoxins*, *immunoglobulins*, *antibodies*, *inhibitors*, *blockers*, *and neutralizers*. The search strategies reported in S1 Text were adapted to accommodate the unique searching features of each database, including database specific MESH and EMTREE controlled vocabulary terms as appropriate (S1 Text). Data was also obtained from FDA briefing documents related to specific agents.

Studies meeting the following criteria were analyzed: employed an *in vivo* animal model challenged with a strain of live *B*. *anthracis*; compared the effects of a therapy designed to inhibit *B*. *anthracis* LT or ET or both to a control agent on survival; and all animals, besides receiving either an anti-toxin or control agent, received similar antibiotic treatment. Three authors, W.X., L.O. and P.Q.E. identified relevant studies. Included studies were searched for additional references. Author consensus resolved uncertainty regarding study inclusion.

### Outcomes examined and data extracted

Data was extracted from included studies using a standardized tool. Authors of included studies were contacted when data required clarification. The primary outcome examined was the effect of anti-toxin therapy on the risk ratio of death based on the duration of survival stipulated in the study. Characteristics of each study extracted included; the species, weight and age of animals studied; the strain, route and dose of *B*. *anthracis* challenge; the type, route, dose, timing and frequency of anti-toxin and control treatment; the type, route, dose, timing and frequency of antibiotic treatment. Other aspects of trial design and conduct extracted included; randomization; blinding; prospective power analyses to determine study group sizes; exposure dose analysis; prospective observation schedules and euthanasia criteria; use of cardiopulmonary supportive measures; secondary endpoints; and industry involvement with a trial. Investigators of analyzed studies were contacted to obtain data not available from the published reports.

### Statistical analysis

The relative risk (RR) of death for anti-toxin treatment versus control was estimated using a random-effects model.[10] Heterogeneity among studies was assessed using the Q statistic and I^2^ value.[11] We first analyzed repeated experiments in each species within each paper, and pooled the data when appropriate. The pooled data were then analyzed by species and by anti-toxin agents to assess their effects. An overall treatment effect was estimated if there was no evidence of species or agent effect. In two groups of experiments, meta-regression was used to assess the relationship between time and treatment effects. We first tested to determine whether there was a significant relationship between treatment time and effect within each study. Then, we pooled the data from both studies and fitted a meta-regression model with a common slope but different intercepts. Sample size calculation was done using STPLAN version 4.5 (https://biostatistics.mdanderson.org/softwaredownload/SingleSoftware.aspx?Software_Id=41) for two-sample binomially distributed outcomes with equal sample sizes in both groups. All analyses were done using R (version 3.3.1) packages *meta* (version 4.3–2) and *metafor* (version 1.9–8).[12,13,14] Two-sided p-values ≤0.05 were considered significant.

## Results

### Search results

**Fig 1:**
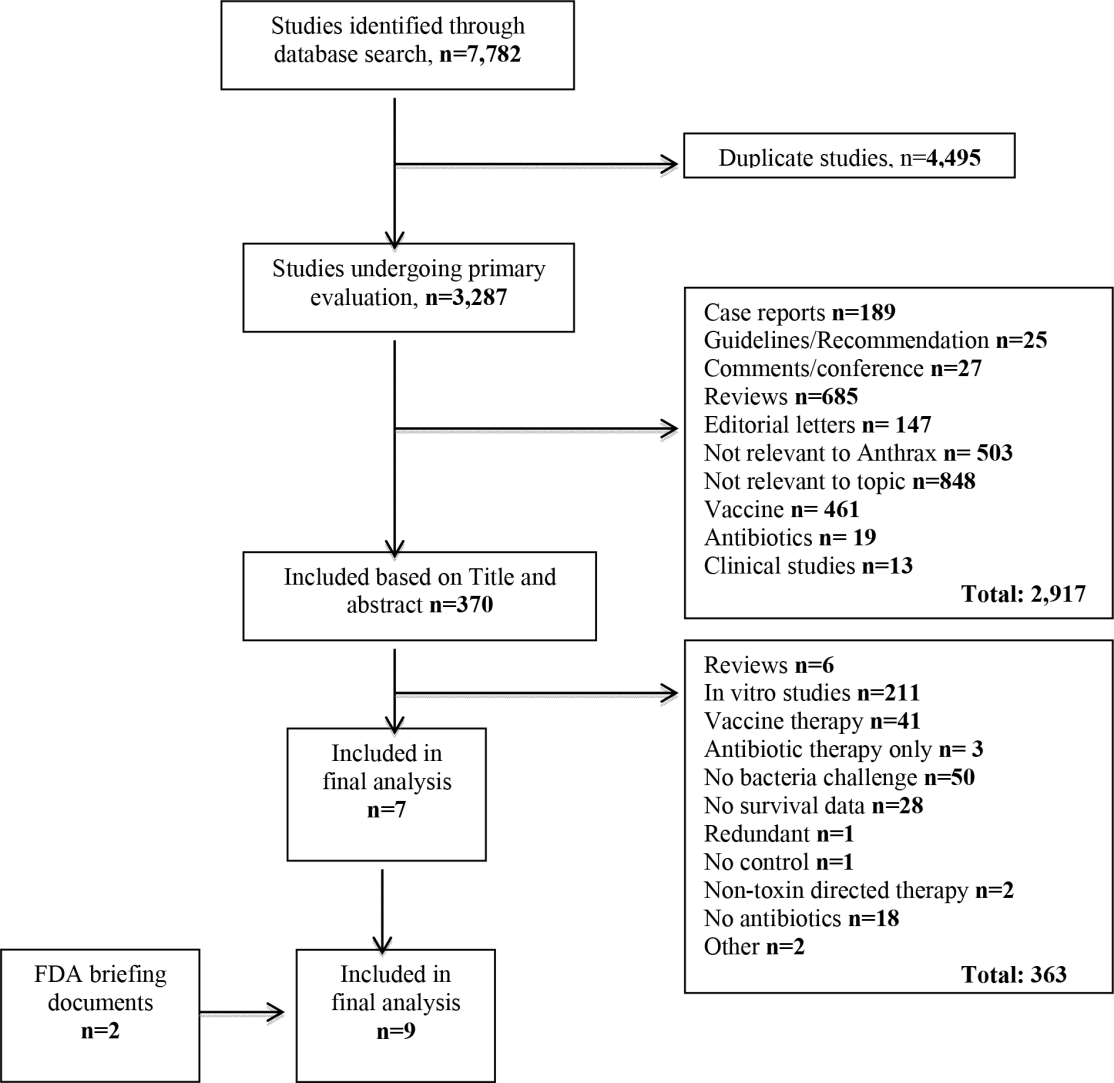
Flow diagram that summarizes results of the literature search.

The literature search identified 7,782 reports for review (Fig 1). Of these, 4,495 were duplicate studies and 2,917 were excluded based on title and abstract review. After full review of the remaining 370 reports, 7 studies were included for analysis (cited in Table 1 and designated in tables by the first author and year of publication). Two FDA briefing documents were also identified which provided data on additional experiments (designated in tables as “FDA” along with the anti-toxin agent tested). Therefore, a total of 9 studies were included in the final analysis.

**Table 1:** Study characteristics (A).

				Infectious Challenge			Antibiotics Treatment				Anti-toxin Treatment				
Author (year)	Exp #	Species	Sex	Type	Route	Dose	Type	Route	Dose	Time (h)	Type	Route	Dose	Time (h)	Trigger
Shoop ('05)	1	Rabbit	NR	Ames	SC	10^4^s	Cipro	SC	5 mg/kg	66	LFI	SC	100 mg/kg	66	NT
Peterson ('06)	2	Mouse	F	Ames	IN	5x10^4^c	Cipro	IP	30 mg/kg	24	AVP	SC	500 ug	24	NT
	3	Gpig	F	Ames	IN	6x10^5^c	Cipro	SC	3.7 mg/kg	24	AVP	IP	5 mg/kg	24	NT
	4	Gpig	F	Ames	IN	6x10^5^c	Cipro	SC	3.7 mg/kg	24	AVP	IP	50 mg/kg	24	NT
	5	Gpig	F	Ames	IN	6x10^5^c	Cipro	SC	7.5 mg/kg	24	AVP	IP	50 mg/kg	24	NT
	6	Gpig	F	Ames	IN	6x10^5^c	Cipro	SC	15 mg/kg	24	AVP	IP	50 mg/kg	24	NT
Migone ('09)	7	Rabbit	M/F	Ames	INH	200xLD50	Levo	Oral	50 mg/kg	0	RAXI	IV	40 mg/kg	NR	PA det
	8	Monkey	M/F	Ames	INH	200xLD50	Cipro	Oral	75 mg	0	RAXI	IV	40 mg/kg	NR	PA det
Migone ('15)	9	Rabbit	M/F	Ames	INH	2.1x10^7^s	Levo	GI, IV	50 mg/kg	84	RAXI	IV	40 mg/kg	84	NT
Kammanadiminti ('14)	10	Rabbit	NR	Ames	INH	2.1x10^7^s	Levo	Oral	50 mg/kg	30	AIGIV	IV	15 U/kg	30	NT
	11	Rabbit	NR	Ames	INH	2.1x10^7^s	Levo	Oral	50 mg/kg	36	AIGIV	IV	15 U/kg	36	NT
	12	Rabbit	NR	Ames	INH	2.1x10^7^s	Levo	Oral	50 mg/kg	48	AIGIV	IV	15 U/kg	48	NT
	13	Rabbit	NR	Ames	INH	2.1x10^7^s	Levo	Oral	50 mg/kg	60	AIGIV	IV	15 U/kg	60	NT
	14	Rabbit	NR	Ames	INH	2.1x10^7^s	Levo	Oral	50 mg/kg	60	AIGIV	IV	15 U/kg	60	NT
	15	Rabbit	NR	Ames	INH	2.1x10^7^s	Levo	Oral	50 mg/kg	72	AIGIV	IV	15 U/kg	72	NT
	16	Rabbit	NR	Ames	INH	2.1x10^7^s	Levo	Oral	50 mg/kg	84	AIGIV	IV	15 U/kg	84	NT
	17	Rabbit	NR	Ames	INH	2.1x10^7^s	Levo	Oral	50 mg/kg	96	AIGIV	IV	15 U/kg	96	NT
	18	Rabbit	NR	Ames	INH	2.1x10^7^s	Levo	Oral	50 mg/kg	96	AIGIV	IV	15 U/kg	96	NT
FDA AIG ('15)	19	Monkey	M/F	Ames	INH	200xLD50	Cipro	Oral	32 mg/kg 16mg/kg	64	AIGIV	IV	15 U/kg	64	NT
	20	Monkey	M/F	Ames	INH	200xLD50	Cipro	Oral	32 mg/kg 16mg/kg	64	AIGIV	IV	30 U/kg	64	NT
Biron ('15)	21	Rabbit	M/F	Ames	INH	150-250xLD50	Doxy	IV	2.0 mg/kg	30	ETI	IV	8mg/kg	30*	PA det
Yamomato ('16)	22	Rabbit	M/F	Ames	INH	200xLD50	Levo	Oral	50 mg/kg	9	ETI	IV	4 mg/kg	9	NT
	23	Rabbit	M/F	Ames	INH	200xLD50	Levo	Oral	50 mg/kg	9	ETI	IM	8mg/kg	9	NT
FDA ETI ('15)	24	Rabbit	NR	Ames	INH	NR	Levo	NR	50 mg/kg	96	ETI	IV	8mg/kg	96	NT
	25	Rabbit	NR	Ames	INH	NR	Levo	NR	50 mg/kg	72	ETI	IV	8mg/kg	72	NT
	26	Rabbit	NR	Ames	INH	NR	Levo	NR	50 mg/kg	30	ETI	IV	16 mg/kg	30	NT
	27	Rabbit	NR	Ames	INH	NR	Levo	Oral	6.5 mg/kg	72	ETI	IV	16 mg/kg	72	NT
	28	Monkey	NR	Ames	INH	NR	Cipro	Oral	10 mg/kg	48	ETI	IV	8mg/kg	48**	PA det
	29	Monkey	NR	Ames	INH	NR	Cipro	Oral	10 mg/kg	48	ETI	IV	8mg/kg	48**	PA det

*If no PA detected then treatment given at 30h post-exposure

**PA detection equivalent to 48h post-exposure

AIGIV- Anthrax Immune Globulin Intravenous; AVP-human monoclonal antibody; c-Colony Forming Units; Cipro-Ciprofloxacin; Doxy- Doxycycline; ETI-ETI-204; Exp #-experiment number; GI-gastrointestinal; IM-intramuscular; IN-intranasal; IP-intraperitoneal; IV-intravenous; LD-lethal dose; Levo- Levofloxacin; LFI-lethal factor inhibitor; NR-not recorded; NT-no trigger; PA det- protective antigen detection; RAXI-Raxibacumab; s-spores; SC-subcutaneous.

### Study characteristics and designs

In the 9 studies reviewed there were a total of 29 individual experiments (i.e., 29 comparisons between an anti-toxin agent and a control treatment under the same conditions), each of which examined 1 of 5 anti-toxin agents including; a hydroxamate lethal factor inhibitor (LFI)– 1 experiment; AVP-21D9, a PA directed monoclonal antibody– 5 experiments; Raxibacumab– 3 experiments; AIG– 11 experiments; and ETI-204–9 experiments (Table 1; for reference, experiments are numbered based on chronology and anti-toxin type). LFI was studied in a rabbit model, AVP-21D9 in mouse and guinea pig models, and the other three agents in both rabbit and cynomolgus monkey models. All models employed the Ames strain of *B*. *anthracis* as the bacteria challenge. These challenges were administered at varying doses and via subcutaneous (SC), intra-nasal (IN) or inhalational (INH) routes. The antibiotic regimens employed in the models included ciprofloxacin, levofloxacin or doxycycline administered via SC, intraperitoneal (IP), intravenous (IV), gastric (GI) or oral routes. Antibiotics were administered anywhere from the time of to 96h following *B*. *anthracis* challenge. Antitoxin agents were delivered via SC, IP, or IV routes and were also administered anywhere from 0 to 96h after bacteria challenge. In some cases, the timing of antibiotic and anti-toxin treatment was scheduled and in others it was based on a change in body temperature or detection of PA in the blood. In Experiment 21, antitoxin was administered at 30h if PA had not yet become detectable. In two Experiments 28 and 29, PA was detected at 48h in animals, and this was the time treatment was administered.

The treatments that control and anti-toxin animals received are shown in Table 2. In some studies, whether and what placebo was used in controls to match the antitoxin agent studied was not available. Table 2 also shows the number of animals that were originally infected in studies and the number that were ultimately randomized or assigned to control and anti-toxin groups and included in analysis for each of the 29 experiments. Altogether, there were 1,283 animals infected with bacteria, of which 748 animals survived to be randomized or assigned; 377 to anti-toxin groups and 371 to a control groups. These 748 animals were included in the present analysis. Finally, Table 2, shows whether the data employed for analysis was available from published reports in a scientific journal alone, from an FDA briefing document alone, or from both. In all cases where data was available from both sources, it was the same.

**Table 2:** Summary of treatments, data sources and numbers of animals challenged and randomized or assigned in experiment.

Author (year)	Experiment Number	Treatments		Data Source	Animal Number		
					Challenged^##^	Randomized or Assigned	
		Control	Antitoxin			Control	Antitoxin
Shoop ('05)	1	Cipro+placebo*	Cipro+LFI	PR	11	4	4
Peterson ('06)	2	Cipro**	Cipro+AVP	PR	20	10	10
	3	Cipro**	Cipro+AVP	PR	18	9	9
	4	Cipro**	Cipro+AVP	PR	10	5	5
	5	Cipro**	Cipro+AVP	PR	10	5	5
	6	Cipro**	Cipro+AVP	PR	10	5	5
Migone ('09)	7	Levo+placebo*	Levo+Raxi	FDA-BD	40	20	20
	8	Cipro+placebo*	Cipro+Raxi	FDA-BD	28	14	14
Migone ('15)	9	Levo+Raxi-buffer	Levo+Raxi	PR	180	37	39
Kammanadiminti ('14)	10	Levo+IGIV	Levo+AIG	PR	16	8	8
	11	Levo+IGIV	Levo+AIG	PR	16	8	7
	12	Levo+IGIV	Levo+AIG	PR	16	8	8
	13	Levo+IGIV	Levo+AIG	PR	16	8	8
	14	Levo+IGIV	Levo+AIG	PR	20	10	8
	15	Levo+IGIV	Levo+AIG	PR	72	20	23
	16	Levo+IGIV	Levo+AIG	PR	19	9	10
	17	Levo+IGIV	Levo+AIG	PR	72	8	7
FDA AIG ('15)	18	Levo+IGIV	Levo+AIG	FDA-BD	336	33	31
	19	Cipro+IGIV	Cipro+AIG	FDA-BD	20	12^#^	12^#^
	20	Cipro+IGIV	Cipro+AIG	FDA-BD	20	12^#^	14^#^
Biron ('15)	21	Doxy+Saline	Doxy+ETI	PR	20	10	10
Yamomato ('16)	22	Levo**	Levo+ETI	PR	21	12	9
	23	Levo**	Levo+ETI	PR	21	12	9
FDA ETI ('15)	24	Levo**	Levo+ETI	FDA-BD	32	5	4
	25	Levo**	Levo+ETI	FDA-BD	32	9	11
	26	Levo**	Levo+ETI	FDA-BD	40	20	20
	27	Levo**	Levo+ETI	FDA-BD	103	38	34
	28	Cipro**	Cipro+ETI	FDA-BD	32	13	13
	29	Cipro**	Cipro+ETI	FDA-BD	32	13	14

*Placebo noted but not described

**No Placebo described

^#^Animals that had not died but were bacteremic at 64h

^##^Number of animals infected at the outset of the experiment, from which some expired prior to later treatment in several experiments

AVP-human monoclonal antibody; Cipro-ciprofloxacin; FDA-BD-Food and Drug Administration briefing document; IGIV- Human Immune Globulin Intravenous; Levo-levofloxacin; LFI-lethal factor inhibitor; PR-published results; Raxi-Raxibacumab.

While all 29 experiments except 1 were randomized, only 5 were blinded ones (Table 3). Twenty-three experiments reported using prospective sample size determinations, 20 used prospective observation schedules and 20 used prospective euthanasia criteria. No study provided hemodynamic or respiratory support for animals. The primary endpoint was survival in all studies and no study included secondary endpoint data such as the effects of anti-toxin treatment on blood pressure, oxygen exchange or other measures of organ function. All experiments accept one (Experiment 21) were conducted by or in association with industry producers of the agents being tested.

**Table 3:** Study designs.

Author (year)	Exp	Agent	Species	Rand.	Blind.	Pro. samp. size	Pro. obs. sched.	Pro. euth. crit.	CP sup.	Prim. endpt.	Sec. Endpt.
Shoop ('05)	1	LFI	Rabbit	Yes	Yes	NR	NR	NR	No	Surv	NR
Peterson ('06)	2	AVP	Mouse	Yes	No	NR	NR	NR	No	Surv	NR
	3	AVP	Gpig	Yes	No	NR	NR	NR	No	Surv.	NR
	4	AVP	Gpig	Yes	No	NR	NR	NR	No	Surv.	NR
	5	AVP	Gpig	Yes	No	NR	NR	NR	No	Surv.	NR
	6	AVP	Gpig	Yes	No	NR	NR	NR	No	Surv.	NR
Migone ('09)	7	RAXI	Rabbit	Yes	Yes	Yes	NR	NR	No	Surv.	NR
	8	RAXI	Monkey	Yes	Yes	Yes	NR	NR	No	Surv.	NR
Migone ('15)	9	RAXI	Rabbit	Yes	Yes	Yes	NR	NR	No	Surv.	NR
Kammanadiminti ('14)	10	AIG	Rabbit	Yes	No	Yes	Yes	Yes	No	Surv.	NR
	11	AIG	Rabbit	Yes	No	Yes	Yes	Yes	No	Surv.	NR
	12	AIG	Rabbit	Yes	No	Yes	Yes	Yes	No	Surv.	NR
	13	AIG	Rabbit	Yes	No	Yes	Yes	Yes	No	Surv.	NR
	14	AIG	Rabbit	Yes	No	Yes	Yes	Yes	No	Surv.	NR
	15	AIG	Rabbit	Yes	No	Yes	Yes	Yes	No	Surv.	NR
	16	AIG	Rabbit	Yes	No	Yes	Yes	Yes	No	Surv.	NR
	17	AIG	Rabbit	Yes	No	Yes	Yes	Yes	No	Surv.	NR
FDA AIG ('15)	18	AIG	Rabbit	Yes	Yes	Yes	Yes	Yes	No	Surv.	NR
	19	AIG	Monkey	Yes	No	Yes	Yes	Yes	No	Surv.	NR
	20	AIG	Monkey	Yes	No	Yes	Yes	Yes	No	Surv.	NR
Biron ('15)	21	ETI	Rabbit	Yes	No	Yes	Yes	Yes	No	Surv.	NR
Yamomato ('16)	22	ETI	Rabbit	Yes	No	Yes	Yes	Yes	No	Surv.	NR
	23	ETI	Rabbit	Yes	No	Yes	Yes	Yes	No	Surv.	NR
FDA ETI ('15)	24	ETI	Rabbit	Yes	No	Yes	Yes	Yes	No	Surv.	NR
	25	ETI	Rabbit	Yes	No	Yes	Yes	Yes	No	Surv.	NR
	26	ETI	Rabbit	Yes	No	Yes	Yes	Yes	No	Surv.	NR
	27	ETI	Rabbit	Yes	No	Yes	Yes	Yes	No	Surv.	NR
	28	ETI	Monkey	Yes	No	Yes	Yes	Yes	No	Surv.	NR
	29	ETI	Monkey	Yes	No	Yes	Yes	Yes	No	Surv.	NR

Blind.–blinding; CP sup–cardiopulmonary support; Euth. crit.–prospective euthanasia criteria; Exp. #—experiment order number; NR–not reported; Prim. endpt.–primary endpoint; Pro. obs. sched.–prospective observation schedule; Pro. samp. size–Prospective sample size analysis; Random.–randomization; Sec. endpt.–secondary endpoint; Surv.—survival

### Effect of anti-toxin treatments on mortality

**Fig 2:**
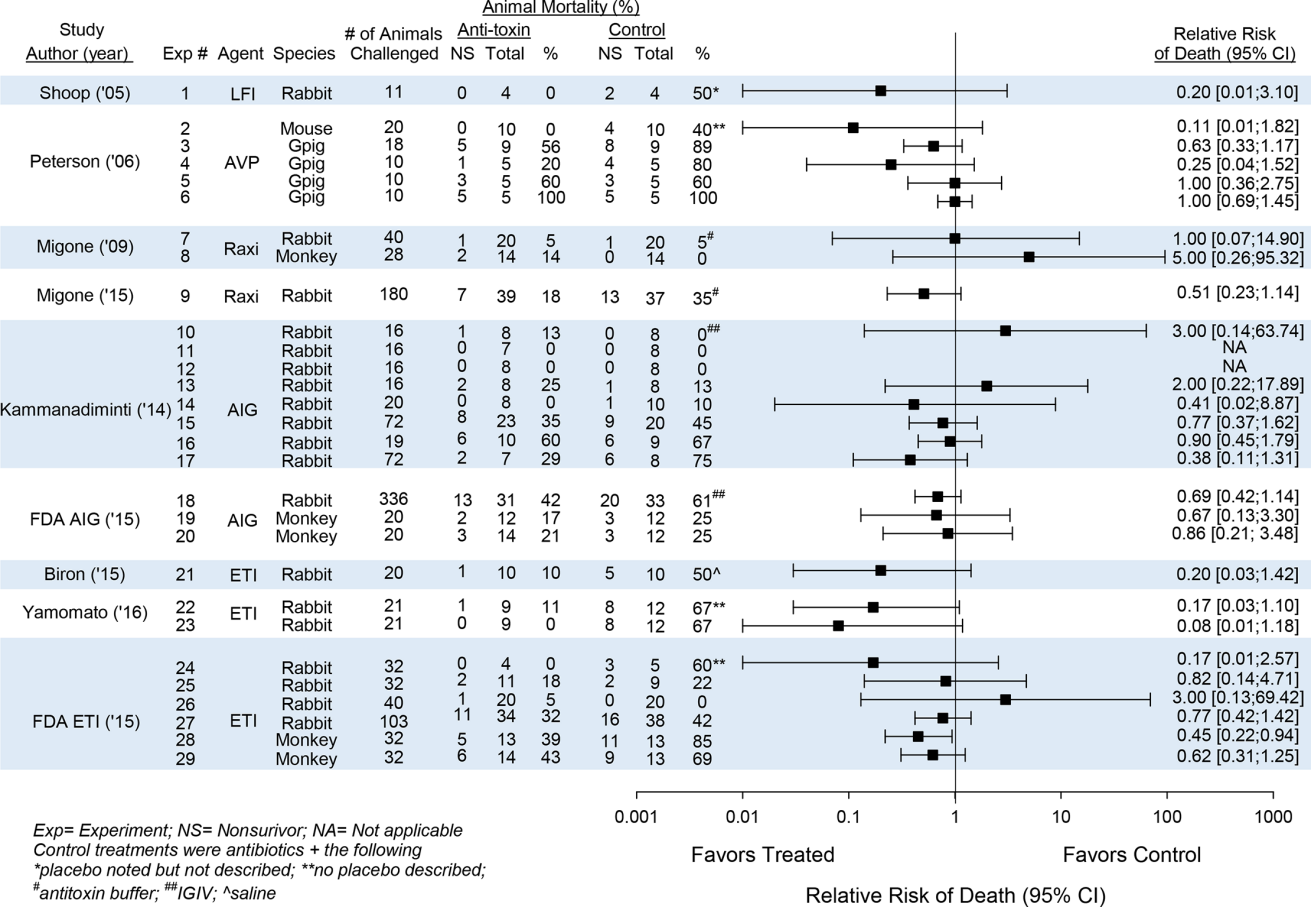
This figure shows the anti-toxin agent and species studied, the number of animals initially infected, the numbers of total and non-surviving animals in treatment and control groups with respective mortality rates, and the effects of the anti-toxin agent (i.e. agents) on the relative risk of death (95%CI) for 29 individual experiments from 9 studies. The anti-toxin agents studied included: lethal factor inhibitor (LFI), AVP-21D9 (AVP), Raxibacumab (Raxi), Anthrax Immune Globulin (AIG), and ETI-204 (ETI). Control treatments were an antibiotic + the following; *a placebo but the placebo was not described; **no placebo was described; ^#^anti-toxin buffer; ^##^human intravenous globulin; ^@^saline (see Table 2).

Survival data with numbers of non-survivors and total numbers of animals in control and anti-toxin groups, percentage mortality rates and relative risks of death (95% CI) are shown for each experiment within their respective studies in Fig 2. In only one experiment did an anti-toxin agent (ET-204 in cynomolgus monkeys, experiment 28) demonstrate a beneficial effect on survival that reached statistical significance, and this experiment was not blinded. However, a total of 20 experiments had effects on the side of benefit and only 4 had effects on the side of harm.

**Fig 3:**
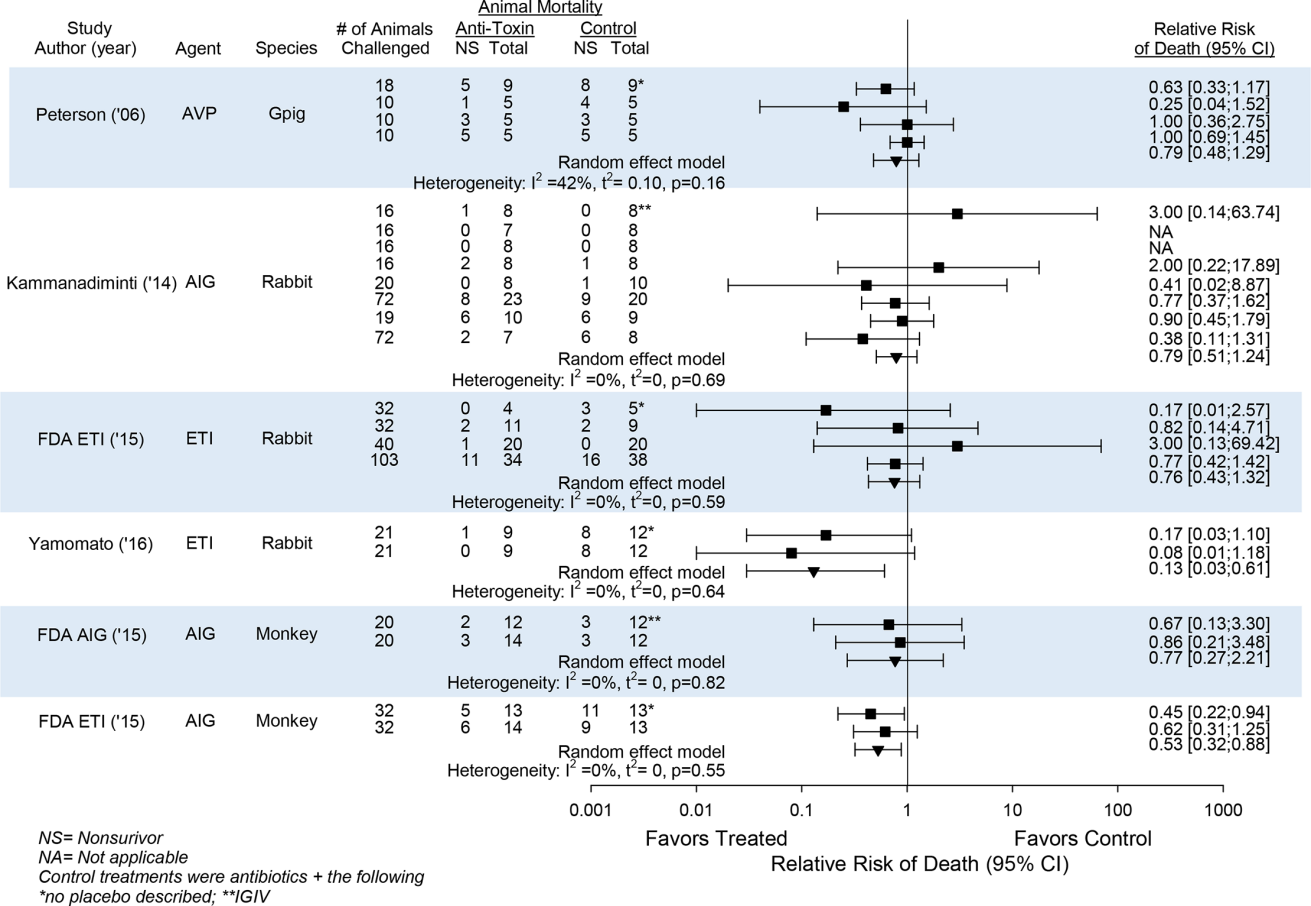
This figure shows data from the six studies testing an anti-toxin agent in more than one experiment in the same species, as well as the overall effects anti-toxin agents had on the relative risk of death (95%CI) (RR) across each of these 6 groups of experiments and the I^2^ and its level of significance for the consistency of these overall effects. Individual RRs for experiments are shown by the squares and overall RRs are shown by the inverted triangles. In the six studies shown, AVP-21D9 (AVP) was studied in four experiments in the guinea pig (Gpig); Anthrax Immune Globulin (AIG) in eight experiments in the rabbit and two experiments in the monkey; and ETI-204 (ETI) in two experiments in the rabbit and two in the monkey. In the five studies testing AIG or ETI, these agents had very consistent effects on the side of benefit across experiments (I^2^ = 0, p≥0.56) in the same species. In the four experiments testing AVP-21D9 in guinea pigs, the RR was also on the side of decreasing mortality but the I^2^ value was moderate although not significant. Control treatments were antibiotics + the following; *no placebo was described; **human intravenous globulin (see Table 2).

Notably, in five of the six studies testing an agent in more than one experiment in the same species, anti-toxin agents had highly consistent effects on the side of decreasing the overall relative risk of death (95%CI) across experiments as follows; 8 experiments with AIG in rabbits, [0.79 (0.51, 1.24), I^2^ = 0, p = 0.69]; 2 experiments with AIG in cynomolgus monkeys, [0.77 (0.27, 2.21); I^2^ = 0, p = 0.82]; 2 experiments with ETI-204 in cynomolgus monkeys, [0.53(0.32, 0.88); I^2^ = 0, p = 0.55]; 4 experiments with ETI-204 in rabbits, [0.76(0.43, 1.32); I^2^ = 0, p = 0.59]; and 2 other experiments with ETI-204 in rabbits, [0.13(0.03, 0.61); I^2^ = 0, p = 0.64] (Figs 2 and 3). In the remaining study with four experiments testing AVP-21D9 in guinea pigs, the relative risk of death (95%CI) was also on the side of decreasing mortality [0.79 (0.48, 1.29)] but the I^2^ value was moderate although not significant (I^2^ = 42%, p = 0.16).

**Fig 4:**
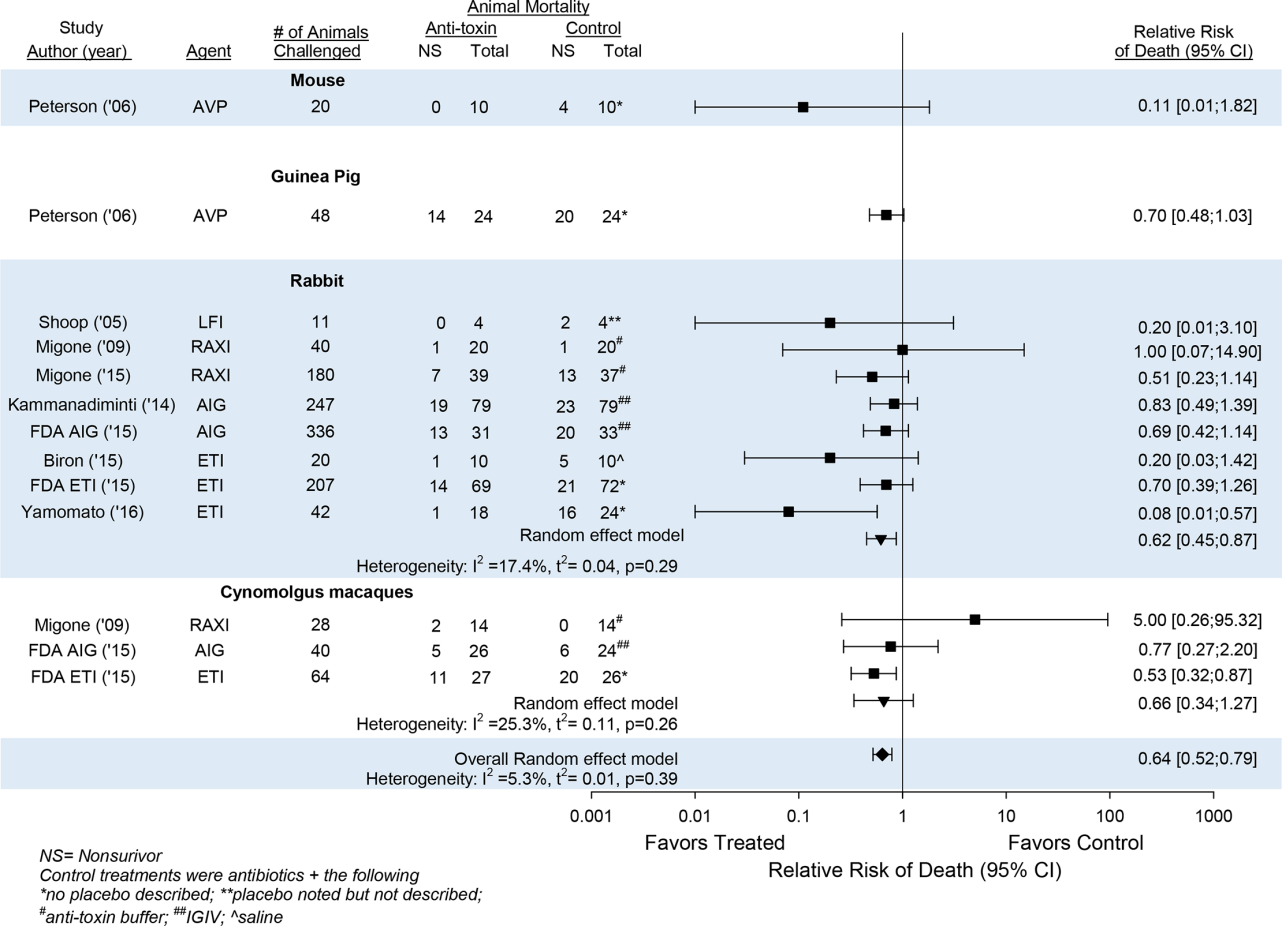
Based on the similarity of the effects of individual anti-toxin agents across experiments in the same species (shown in Fig 3), this figure shows the overall effects of the anti-toxin agents on the relative risk of death (95%CI) (RR) across studies within the same species and the I^2^ and its level of significance for the consistency of these overall effects. Individual RRs for studies are shown by the squares and overall RRs are shown by the inverted triangles. AVP-21D9 in one study in mice and one study in guinea pigs, LFI, Raxibacumab, AIG and ETI-204 in eight studies in rabbits and Raxibacumab, AIG and ETI-204 in three studies in monkeys were all associated with reductions in RR. Because the effects of the anti-toxin agents were consistent across these studies in the same species (I^2^≤25.3%), the effects of treatment on the RR averaged across all four anti-toxin agents and all four species is shown by the diamond at the bottom of the figure. Control treatments were antibiotics + the following; *no placebo was described; **placebo was noted but not described; ^#^anti-toxin buffer; ^##^human intravenous globulin; ^@^saline (see Table 2).

Based on the similarity of the effects of individual anti-toxin agents across experiments in the same species, we further analyzed the overall effects of the agents across studies within the same species (Fig 4). In this analysis, all agents were associated with a decrease in the relative risk of death (95%CI) as follows; AVP-21D9 in one study in mice [0.11(0.01, 1.82)]; AVP-21D9 in one study in guinea pigs [0.70(0.48, 1.03)]; LFI, Raxibacumab, AIG and ETI-204 in a total of eight studies in rabbits [0.62(0.45, 0.87); I^2^ = 17.4%, p = 0.29]; and Raxibacumab, AIG and ETI-204 in a total of three studies in cynomolgus monkeys [0.66(0.34, 1.27); I^2^ = 25.3%, p = 0.26].

Because the effects of the anti-toxin agents were consistent across these studies in the same species (I^2^≤25.3%), we then examined the effects of anti-toxin treatments across all four agents and all four species (Fig 4). In this analysis, antitoxin agents decreased the overall relative risk of death (95%CI) significantly [0.64(0.52, 0.79)] and in a pattern that was consistent across agents and species (I^2^ = 5.3%, p = 0.39).

The prior systematic review observed that anti-toxin agents demonstrated greater benefit when administered later in antibiotic treated models.[8] Consistent with that observation, in the two studies here which administered anti-toxin agents earlier in some experiments and later in others in the same species (AIG in experiments 10 to 17 and ETI-204 in experiments 24 to 27, both in rabbits) later treatment appeared associated with greater decreases in the relative risk of death. The windows of treatment examined within each set of experiments extended from 30 to 96h following challenge. Although these relationships between time and the effects of anti-toxin agents on the log relative risk of death [slope in log(RR) (95% CI)] did not reach significance for either AIG [-0.023 (-0.060, 0.015), p = 0.24] or ETI-204 [-0.042 (-0.104, 0.021), p = 0.19] alone, the trend for the two agents when combined approached significance [-0.028 (-0.060, 0.005), p = 0.09].

### Study sizes necessary to confirm the beneficial trends noted with raxibacumab in experiment 9 and AIG in experiments 18

The two largest experiments testing Raxibacumab and AIG (180 and 336 initially infected animals respectively), showed that these agents when combined with antibiotics during established *B*. *anthracis* infections produced beneficial trends but not significant increases in survival. Based on the numbers of animals that were originally infected, the number available at the time of randomization, and the treatment effects observed in these prior experiments (Table 2 and Fig 2), we determined the study sizes necessary to independently show that each of these agents would result in a significant improvement in survival. In the case of Raxibacumab, 104 animals per group would need to be randomized to control and anti-toxin groups to have 80% power to detect a difference of 17% in mortality (35% vs 18%) at a 2-sided significance level of 0.05. Based on the 58% mortality rate observed prior to randomization and treatment in Experiment 9, in this proposed study 496 animals would therefore have to be initially infected. Applying data similarly from Experiment 18 with AIG (81% mortality prior to randomization), 1138 animals would need to be infected to provide 108 animals per group to be randomized to control and anti-toxin groups. This number of randomized animals would have an 80% power to detect a difference of 19% in mortality (61% vs 42%) at a 2-sided significance level of 0.05.

## Discussion

In this analysis, only one *B*. *anthracis* anti-agent in a single experiment had a beneficial effect on survival that reached significance (i.e., ETI-204 in a cynomolgus monkey model, experiment 28). However, in 20 other experiments anti-toxin agents had effects on the side of benefit, while in only 4 experiments were these effects on the side of harm. Furthermore, when examined within species and across anti-toxin agents, treatment had consistent beneficial effects in rabbits and cynomolgus monkey models in patterns that were significant or had a significant trend. Finally, when examined across all agents in all species, anti-toxin agents reduced the relative risk of death significantly and consistently.

On the one hand, these findings in antibiotic treated animal models employing live *B*. *anthracis* challenge provide a basis for a therapeutic approach for this infection that incorporates anti-toxin agents. They also add support to the CDC’s recommendation to administer approved anti-toxin agents along with conventional therapies in patients with evidence of severe *B*. *anthracis* infection.[3,4] Failure to achieve significance in all but one of the individual experiments analyzed may relate in part to the difficult conditions under which virulent *B*. *anthracis* strains must be studied and the limitations those constraints put on sample sizes. In addition, antibiotics can be effective treatment for *B*. *anthracis* infection and demonstrating that anti-toxin agents add to those benefits also increases the size and complexity of the studies needed to show significant benefit. Trends towards increased survival with individual agents in this meta-analysis are consistent with some clinical observations that have been made with anti-toxin agents. In contrast to the experience with AIG during the outbreak of soft tissue infection in injection drug users, it has been noted that two of three isolated cases of inhalational disease receiving AIG survived.[8] This survival rate appears greater than might be expected from past experience in patients not receiving anti-toxin therapy. Also, a historical review of inhalational cases presenting in the US since 1900 suggested that use of anti-toxin preparations was protective.[15] In both of these retrospective reports though, authors noted that there were confounding factors potentially contributing to the apparent beneficial effects of anti-toxin agents. It is possible though that anti-toxin agents are more effective in the inhalational form of anthrax infection than in soft tissue disease. As noted in the introduction, FDA approval of anti-toxin agents has been for inhalation infection only.

On the other hand, several aspects of the studies analyzed here weaken conclusions regarding the benefits of this therapeutic approach. As with most of the experiments analyzed, the one individual experiment in which an anti-toxin agent was significantly beneficial was un-blinded. In the five experiments that were blinded, only two were powered to possibly show a convincing treatment effect (n = 76 for Raxibacumab in experiment 9, and n = 64 in experiment 18 with AIG) and in both of these experiments treatment was only associated with trends in improved survival (p = 0.087 and p = 0.135 respectively). Also, different from clinical trials, none of the animal studies provided secondary endpoint data such as serial measures of cardiopulmonary or other organ function to provide a basis and support for survival effects that trended towards but did not reach significance. Although all of the studies analyzed included antibiotic therapy, none of them employed the type of cardiopulmonary support with titrated fluid, vasopressor, oxygen and ventilation therapies that critically ill patients receive. While some animal studies have suggested that anti-toxin agents can augment hemodynamic support in *B*. *anthracis* toxin challenged models, such data is not available in animal models of *B*. *anthracis* infection.[16,17] Failure to include these supportive therapies in infection models probably relates to the fact that just as the virulence of *B*. *anthracis* limits the size of animal experiments testing anti-toxin agents, it also constrains the use of these types of titrated therapies that require invasive interventions and frequent and close measurements to adequately administer. It is known however, that antibiotics and hemodynamic support have synergistic beneficial survival effects during severe bacterial infection.[18,19] Such synergistic effects could lessen or negate the benefit of anti-toxin agents. It has been noted that one of the reasons selective anti-inflammatory agents (e.g., anti-TNF antibodies) were beneficial in preclinical animal studies but not in clinical trials was because the preclinical experience did not account for the types of cardiopulmonary support patients receive.[20] Finally, the results of analysis here, although not reaching statistical significance, suggest that delaying administration of anti-toxin agents and antibiotics following bacterial challenge in these models increased the effectiveness of treatment. Presumably this delay allowed bacteria to replicate and toxin concentrations to increase to pathogenic levels that anti-toxin exerted a beneficial effect on. During an outbreak of *B*. *anthracis* infection, some patients will likely present early, when antibiotics alone are sufficient therapy, while others will present so late that no therapy will be effective. Although it has been proposed that circulating PA or toxin levels may predict patients most likely to benefit from anti-toxin agents, proven methodology to reliably make this prediction is not available.

Ultimately, absence of a clear and significant beneficial effect of anti-toxin agents in most individual experiments in the present analysis may relate to the fact that while toxin production is important in the pathogenesis of *B*. *anthracis* infection, other bacterial components contribute as well. Growing evidence indicates that *B*. *anthracis* cell wall and its peptidoglycan component can produce an injurious and lethal host inflammatory response.[21,22,23] Proteases other than LT may also contribute to tissue injury and organ dysfunction during infection.[24] Therefore, adjunctive therapies targeting toxin production alone, while potentially beneficial, may not have as great an impact on overall outcome as expected. Larger experiments than the ones available for analysis here appear necessary to show conclusively that targeting toxin during active infection improves survival.

Three of the anti-toxin agents we examined including AIG, Raxibacumab and ETI-204, have received FDA approval. AIG and Raxibacumab have also been included in the Strategic National Stockpile, and ETI-204 may be as well. The FDA publishes criteria that must be met during testing to support its approval of agents that can only be investigated in animal models of disease because human efficacy studies are not ethical or feasible, as was the case with these agents.[25] These criteria include among others randomization, blinding and prospective statistical plans, endpoints, euthanasia criteria, and observation schedules. Although many of these criteria were addressed in individual experiments, based on published methods and correspondence with investigators, only one experiment testing AIG in rabbits (Experiment 18), and none of those testing ETI-204 were blinded (Table 3). Absence of blinding weakens the results of studies even when other design criteria are met.

Compared to a prior review of studies examining the effects of antitoxin agents in *B*. *anthracis* challenged and antibiotic treated preclinical models which included 5 studies and 424 animals, the present one analyzed 9 studies and a total of 748 animals.[8] This difference was due to our inclusion of studies assessing LFI and AVP-21D9 and of more recent studies testing ETI-204. Different from the prior analysis, the present one also included a meta-analysis of the retrieved studies.

It has been suggested that with a biologically plausible basis and the limited adverse effects of anti-toxin agents in healthy volunteer safety studies, the beneficial trends anti-toxin agents have demonstrated in animal *B*. *anthracis* infection models provide a basis for their administration clinically.[8] Furthermore, a pressing need to rapidly establish a national stockpile of adjunctive therapies for use during an outbreak of *B*. *anthracis*, may have justified the approval and inclusion of AIG and Raxibacumab based on the studies analyzed here. At present, approved anti-toxin agents should be made available for use in patients with invasive *B*. *anthracis* infection that are not responding to conventional treatment. Although not examined here, there is also data and a rational for the FDA indicated use of anti-toxin agents when the underlying *B*. *anthracis* strain being treated is resistant to available antibiotic therapy or for patients in whom antibiotic therapy is not available or contra-indicated[26,27].

However, for agents that can be tested clinically, it is expected that regardless of biologic plausibility or safety data, they show statistical significance in clinical trials to justify their approval and use. Stocking and maintaining anti-toxin agents in the SNS is costly.[28, 29] Given the weak effects anti-toxin agents demonstrated and the limitations of the studies we have analyzed, it seems very important that additional testing of these agents in adequately powered and designed studies be considered to clearly document their efficacy and support their continued inclusion in the SNS. Ideally, although possibly not practical due to associated risks, these studies would include in addition to antibiotics, the types of conventional cardiopulmonary support and monitoring patients with severe *B*. *anthracis* infection would typically receive. Such studies would be analogous to the phase 4 type testing often required of agents approved based on clinical testing. Clearly such studies would be difficult and expensive. As shown with the sample size calculation in the results, 496 and 1138 animals would need to be challenged to provide sufficient numbers of subsequently randomized animals to confirm that Raxibacumab and AIG respectively would produce significant improvements in survival when added to antibiotics during life-threatening infection. To also test the effects of an anti-toxin agent in groups receiving some sort of titrated cardiopulmonary support would add further to these animal requirements. Despite the expense of such additional studies however, this cost would not be as great as the continued maintenance of a national stockpile of anti-toxin agents that are later found to be ineffective when administered to patients during a widespread outbreak of *B*. *anthracis* infection.

## Supporting information

S1 Text Search terms and strategies. (DOCX)

S2 Text PRISMA checklist for anthrax meta-analysis. (DOCX)
