# Correction: A systematic review and meta-analysis of preclinical trials testing anti-toxin therapies for *B*. *anthracis* infection: A need for more robust study designs and results

**DOI:** 10.1371/journal.pone.0189239

**Published:** 2017-12-04

**Authors:** Wanying Xu, Lernik Ohanjanian, Junfeng Sun, Xizhong Cui, Dante Suffredini, Yan Li, Judith Welsh, Peter Q. Eichacker

The second author's name is spelled incorrectly. The correct name is: Lernik Ohanjanian. The correct citation is: Xu W, Ohanjanian L, Sun J, Cui X, Suffredini D, Li Y, et al. (2017) A systematic review and meta-analysis of preclinical trials testing anti-toxin therapies for *B*. *anthracis* infection: A need for more robust study designs and results. PLoS ONE12(8): e0182879. https://doi.org/10.1371/journal.pone.0182879
