# Supplementary material for: A systematic review and meta-analysis of preclinical trials testing anti-toxin therapies for B. anthracis infection: A need for more robust study designs and results
Source: PLoS One. 2017 Aug 10;12(8):e0182879. doi: 10.1371/journal.pone.0182879 (PMC5552191; doi:10.1371/journal.pone.0182879)
Supplement: S1 Text — (DOCX) [file pone.0182879.s001.docx]

**S1 Text**

**Search terms and strategies**

PubMed

(anthrax[mesh] OR anthrax[tiab] OR bacillus anthracis[mesh] OR “bacillus anthracis”[tiab] OR “b anthracis”[tiab]) AND (antitoxins[mesh] OR antitoxin[tiab] OR antitoxins[tiab] OR immunoglobulins[mesh] OR immunoglobulin[tiab] OR immunoglobulins[tiab] OR “immune globulin”[tiab] OR “immune globulins”[tiab] OR AIGIV[tiab] OR anthrivig[tiab] OR antibodies[mesh] OR antibody[tiab] OR antibodies[tiab] OR “toxin directed”[tiab] OR inhibitor[tiab] OR inhibitors[tiab] OR inhibiting[tiab] OR inhibition[tiab] OR blocker[tiab] OR blockers[tiab] OR blocking[tiab] OR neutralizer[tiab] OR neutralizers[tiab] OR neutralizing[tiab] OR neutralization[tiab] OR raxibacumab[tiab] OR treatment[tiab] OR therapy[tiab]) Filters: Publication date from 2015/01/01

EMBASE

'anthrax'/exp/mj OR anthrax:ab,ti OR 'bacillus anthracis'/exp/mj OR 'bacillus anthracis':ab,ti OR 'b anthracis':ab,ti AND ('antitoxin'/exp OR antitoxin:ab,ti OR antitoxins:ab,ti OR 'immunoglobulin'/exp OR immunoglobulin:ab,ti OR immunoglobulins:ab,ti OR 'immune globulin':ab,ti OR 'immune globulins':ab,ti OR aigiv:ab,ti OR anthrivig:ab,ti OR 'antibody'/exp OR antibody:ab,ti OR antibodies:ab,ti OR 'toxin directed':ab,ti OR inhibitor:ab,ti OR inhibitors:ab,ti OR inhibiting:ab,ti OR inhibition:ab,ti OR blocker:ab,ti OR blockers:ab,ti OR blocking:ab,ti OR neutralizer:ab,ti OR neutralizers:ab,ti OR neutralizing:ab,ti OR neutralization:ab,ti OR 'raxibacumab'/exp OR raxibacumab:ab,ti OR treatment:ab,ti OR therapy:ab,ti) AND [2015-2016]/py

Scopus

( TITLE-ABS ( anthrax  OR  "bacillus anthracis"  OR  "b anthracis" )  AND  TITLE-ABS ( antitoxin  OR  antitoxins  OR  immunoglobulin  OR  immunoglobulins  OR  "immune globulin"  OR  "immune globulins"  OR  aigiv  OR  anthrivig  OR  antibody  OR  antibodies  OR  "toxin directed"  OR  inhibitor  OR  inhibitors  OR  inhibiting  OR  inhibition  OR  blocker  OR  blockers  OR  blocking  OR  neutralizer  OR  neutralizers  OR  neutralizing  OR  neutralization  OR  raxibacumab  OR  treatment  OR  therapy ) )  AND  ( LIMIT-TO ( PUBYEAR ,  2016 )  OR  LIMIT-TO ( PUBYEAR ,  2015 ) )
